# Circulating Levels of Tumor Necrosis Factor-Alpha Receptor 2 Are Increased in Heart Failure with Preserved Ejection Fraction Relative to Heart Failure with Reduced Ejection Fraction: Evidence for a Divergence in Pathophysiology

**DOI:** 10.1371/journal.pone.0099495

**Published:** 2014-06-12

**Authors:** Brendan N. Putko, Zuocheng Wang, Jennifer Lo, Todd Anderson, Harald Becher, Jason R. B. Dyck, Zamaneh Kassiri, Gavin Y. Oudit, on behalf of the Alberta HEART Investigators

**Affiliations:** 1 Division of Cardiology, Department of Medicine, University of Alberta, Edmonton, Alberta, Canada; 2 Mazankowski Alberta Heart Institute, University of Alberta, Edmonton, Alberta, Canada; 3 Libin Cardiovascular Institute, University of Calgary, Calgary, Alberta, Canada; 4 Department of Pediatrics, University of Alberta, Edmonton, Alberta, Canada; 5 Department of Physiology, University of Alberta, Edmonton, Alberta, Canada; Emory University, United States of America

## Abstract

**Background:**

Various pathways have been implicated in the pathogenesis of heart failure (HF) with preserved ejection fraction (HFPEF). Inflammation in response to comorbid conditions, such as hypertension and diabetes, may play a proportionally larger role in HFPEF as compared to HF with reduced ejection fraction (HFREF).

**Methods and Results:**

This study investigated inflammation mediated by the tumor necrosis factor-alpha (TNFα) axis in community-based cohorts of HFPEF patients (n = 100), HFREF patients (n = 100) and healthy controls (n = 50). Enzyme-linked immunosorbent assays were used to investigate levels of TNFα, its two receptors (TNFR1 and TNFR2), and a non-TNFα cytokine, interleukin-6 (IL-6), in plasma derived from peripheral blood samples. Plasma levels of TNFα and TNFR1 were significantly elevated in HFPEF relative to controls, while levels of TNFR2 were significantly higher in HFPEF than both controls and HFREF. TNFα, TNFR1 and TNFR2 were each significantly associated with at least two of the following: age, estimated glomerular filtration rate, hypertension, diabetes, smoking, peripheral vascular disease or history of atrial fibrillation. TNFR2 levels were also significantly associated with increasing grade of diastolic dysfunction and severity of symptoms in HFPEF.

**Conclusions:**

Inflammation mediated through TNFα and its receptors, TNFR1 and TNFR2, may represent an important component of a comorbidity-induced inflammatory response that partially drives the pathophysiology of HFPEF.

## Introduction

Heart failure (HF) continues to have a significant clinical burden with a high mortality and morbidity, and has a worse one-year prognosis than cancer [1]. There are several classification schemes in HF; however, a common clinical approach involves classifying ambulatory patients with chronic HF into HF with preserved (≥50%) or reduced left-ventricular ejection fraction (<50%; HFPEF or HFREF, respectively) [1], [2]. HFPEF presently accounts for approximately 40% of HF diagnoses, with a rising incidence, and mortality and morbidity comparable to HFREF [3]–[5]. Despite similarities in outcomes, it appears that HFPEF and HFREF represent distinct groups, with many pathophysiological differences, along the continuum of the HF [6]. Indeed, differential responses to therapeutic interventions between these two groups strongly support this concept. Clinical trials have validated angiotensin-converting enzyme inhibitors (ACEi), angiotensin II-receptor blockers (ARB), beta-adrenergic receptor antagonists (beta-blockers) and mineralocorticoid receptor antagonists (MRA) as therapeutics in HFREF [1]. Conversely, trials of ACEi, ARB, beta-blockers, MRA and phosphodiesterase-5 inhibitor failed to show any consistent and significant improvement in the clinical outcomes of patients with HFPEF.

Biomarker studies have differentially implicated various pathways in HF, including fibrosis and extracellular matrix remodeling, oxidative and cardiomyocyte stress, and inflammation [7]–[12]. A recent report showed that circulating tumor necrosis factor-alpha (TNFα) receptor 1 (TNFR1) levels are significant predictors of incident HF, in particular for HFPEF versus HFREF [13]. Circulating TNFα and its two receptors (TNFR1 and TNFR2) are elevated in patients with heart failure relative to controls [6], [14]. The study presented herein expands on previous work by exploring associations between diastolic dysfunction or heart failure severity, and plasma levels of TNFα, TNFR1 and TNFR2, as well as a non-TNFα family cytokine, interleukin-6 (IL-6), in community-based cohorts of healthy controls, and ambulatory HFPEF and HFREF patients. Using well-defined HF cohorts, we set out to elucidate some elements of the mechanisms that drive HFPEF and HFREF. Our novel findings suggest that elevated plasma levels of TNFα receptors, in particular TNFR2, are more closely linked to the pathophysiology of HFPEF than HFREF.

## Methods

### Ethics statement

This study conforms with the conventions outlined in the Declaration of Helsinki, it received internal ethics board approval at the Universities of Alberta and Calgary for use of human subjects, and all subjects gave written informed consent [15].

### Patient recruitment and baseline analysis

As part of a prospective clinical study, the Alberta HEART (Heart Failure Etiology and Analysis Research Team) project, community-based, ambulatory patients with clinical diagnoses of HF and healthy age- and gender-matched controls (n = 50) were consecutively recruited for comprehensive clinical, echocardiographic and biomarker analyses during a three-year period beginning in 2010 through the end of 2012. Blood pressure was recorded sitting or recumbent, and detailed clinical data were compiled at the time of enrollment. Transthoracic echocardiograms were performed using the Phillips IE33 ultrasound platform. Echocardiograms were interpreted by cardiologists with specialized echocardiography training who were blinded to both the clinical classification and biomarker analyses. LVEF was assessed using Simpson's biplane method of disks. HF patients were adjudicated as HFREF (n = 100) or HFPEF (n = 100) using an LVEF cutoff of 50%, according to clinical practice guidelines [1], [2]. Adjudication of New York Heart Association (NYHA) functional class and primary etiology of HF were determined by cardiologists blinded to biomarker analyses. Grading of diastolic dysfunction was performed by blinded members of the authorship team based on previously published guidelines [16]. LA volume index, lateral e' and medial e' were used as a binary classifier for diastolic dysfunction, after which E/A ratio, or average E/e' ratio, for patients in AFib, was used to ascertain grade in those determined to have diastolic dysfunction. Diastolic dysfunction analyses could not be performed due to poor echocardiographic visualization in 9 HFPEF patients and 14 HFREF patients, and a further 1 HFPEF patient and 7 HFREF patients were excluded from diastolic function analyses due to the presence of severe mitral regurgitation (MR).

BNP levels were assessed as previously described using an Alere Triage reagent pack (Alere Inc., Ottawa, ON, CAN) read in an automated DxI 800 immunoanalyzer (Beckman-Coulter, Fullerton, CA, USA) at provincial heath laboratories in the province of Alberta [17]. Serum levels of creatinine and lipid profiles were available in study subjects' health records as part of their clinical evaluation. Inclusion criteria for healthy controls were as follows: no history of cardiovascular or renal disease, hypertension, diabetes, or atrial fibrillation; and no prescriptions for antiarrhythmics; ACEi; ARB; beta-blockers; digoxin; loop or thiazide diuretics; or MRA. Exclusion criteria for all study groups were any of the following: age less than 18 years; known malignancy, with expected survival time less than one year; pregnancy within the previous six months; recent cardiac event, including acute MI and decompensated HF; moderate or severe pulmonary hypertension; or severe mitral or aortic valvular stenosis.

### Plasma biomarker analyses

Blood for plasma analysis was collected during a one-day baseline enrolment which included the acquisition of the echocardiograms and ECGs. Patients were rested and sitting while blood was collected into cooled lithium-heparin tubes, which were immediately placed on ice prior to plasma fractionation and deep freezing at ≤−75°C. Commercially-available enzyme-linked immunosorbent assay (ELISA) kits for TNF-α, TNFR1, TNFR2 and IL-6 (catalogue no. 's STA00C, SRT100, SRT200 and S6050, respectively, R&D Systems, MN, USA) were used as previously described [18], [19]. Absorbance was measured at 450 nm with the wavelength correction set to 540 nm for all assays using a SpectraMax M5 Plate Reader (Molecular Devices, CA, USA). Detection rates for the ELISAs were 72.4%, 100%, 100% and 81.2%, respectively. The inter- and intra-assay coefficients of variation were 13.1% and 9.5% (n = 4); 7.0% and 5.2% (n = 4); 5.3% and 3.5% (n = 4); and 8.0% and 6.3% (n = 4), respectively.

Plasma ACE2 activity was measured using a previously established immunofluorescence protocol [20]. Lithium heparin anti-coagulated platelet-free plasma samples were diluted to a final ratio of 30∶70 in plasma assay buffer: 1 M NaCl (Sigma Chem. Co., MO, USA), 75 mM Tris-HCl (Invitrogen, CA, USA) and 5 mM ZnCl_2_ (Sigma Chem. Co., MO, USA) at pH 6.5. Assay buffer was made to contain various protease inhibitors: 10 µM captopril (ACE inhibitor; Sigma Chem. Co., MO, USA); 5 µM amastatin (aminopeptidase inhibitor; Sigma Chem. Co., MO, USA); and Protease Inhibitor Cocktail (Sigma Chem. Co., MO, USA) dissolved to achieve a concentration of 10 µM bestatin (aminopeptidase inhibitor), 1 µM E-64 [N-(trans-Epoxysuccinyl)-L-leucine-4-guanidinobutylamide; cysteine protease inhibitor], 770 nM pepstatin A (aspartyl protease inhibitor), 154 µM AEBSF [4-(20aminoethyl)benzenesulfonyl fluoride hydrochloride; serine protease inhibitor], 75 nM phosphoramidon (neprilysin inhibitor), 15 nM aprotinin (serine protease inhibitor) and 75 nM leupeptin (cysteine, serine and threonine proteases inhibitor). The use of protease inhibitors in the buffer solution was meant to ensure that the fluorogenic substrate and specific ACE2 inhibitor (both described below), which are integral to the assay, were not cleaved by other proteases naturally present in human plasma. Samples were incubated with Dnp-quenched Mca-containing fluorogenic substrate (catalogue no. ES007, R&D Systems, MN, USA) at a final concentration of 10 µM at 37°C. To find the fluorescence increase due to ACE2 activity, plasma samples were assayed both in the presence and absence of the specific linear ACE2 inhibitor, DX600 (catalogue no. 002-26, Phoenix Pharmaceuticals, CA, USA). Fluorescence was measured with excitation and emission settings at 320 nm and 405 nm, respectively, using SpectraMax M5 plate reader (Molecular Devices, CA, USA). The maximal fluorescence increase due to ACE2 activity was determined from the maximum fluorescence difference between inhibited and uninhibited aliquots; this was normalized to a standard curve for Mca-containing fluorescent peptide (catalogue no. M-1975, Bachem, CA, USA) and scaled for time of measurement and plasma volume over 24 hours, with 1 hour as the baseline. All ACE2 enzymatic activity values herein are expressed in pmol/hr/mL, which describes the amount of substrate turned over per unit time per unit volume of plasma. Inter- and intra-assay coefficients of variation were 10.2% and 7.5% (n = 10), respectively.

### Data analysis

Continuous data in tables are expressed as median with interquartile range (IQR) in parentheses. Continuous data in figures are expressed as box and whisker plots on a logarithmic scale to account for non-normal distributions, where boxes represent IQR with median as a bisecting line, while whiskers represent the minimum and maximum values. Categorical data are expressed as percentages. All categorical data were compared using Pearson Chi-square tests, while continuous variables analyzed by category were compared using Mann-Whitney U Test or Kruskal-Wallis Test with Mann-Whitney U Test for pairwise comparisons, where appropriate. Associations between categorical factors or continuous covariates and circulating inflammatory markers were tested using binary logistic or linear regression analyses, respectively, with log-transformed inflammatory marker levels to account for non-normal distributions. Associations between increasing grade of diastolic dysfunction or NYHA functional class and plasma biomarkers were tested using ordinal logistic regression analyses with log-transformed inflammatory marker levels. Forest plots are used to graphically display results from logistic regression analyses. A p-value<0.05 was considered significant for all statistical analyses. Statistical analyses were conducted using SPSS Statistics Version 20 (IBM, NY, USA). Obesity was defined as BMI≥30 kg/m^2^ per WHO criteria [21]. Estimated glomerular filtration rate (eGFR) was calculated by the revised four-variable Modification of Diet in Renal Disease equation [22]. Left-ventricular hypertrophy (LVH) was defined as left-ventricular mass index (LVMI) ≥115 g/m^2^ for males and ≥95 g/m^2^ for females per previously published criteria [23].

## Results

### Baseline clinical profile and assessments of heart function

Demographic and clinical information for the control, HFPEF and HFREF groups is displayed in Table 1. In this study, the HF patient populations recapitulated observations in previous reports [3]–[5], [24]. The study subjects were predominantly of white race in all study groups (Table 1). HFPEF patients were older (p = 0.003), and significantly more were obese (p = 0.016) and hypertensive than HFREF patients, while neither HF group had different gender ratios (p = 0.210), histories of diabetes, peripheral vascular disease (p = 0.234) or atrial fibrillation (AFib; Table 1). Interestingly, total cholesterol (p = 0.774), triglycerides (p = 0.656) and total cholesterol:high-density lipoprotein (HDL) ratio (p = 0.187) were not significantly different between HF phenotypes, despite differences in obesity prevalence (Table 1). Ischemic etiology was more prevalent in HFREF than HFPEF, but both groups were on evidence-based therapeutic regimens (Table 1). Importantly, drugs that can affect inflammation were comparably used by the two HF groups: NSAID use was equivalent and statin use was not significantly different (p = 0.438; Table 1). NYHA functional class distribution was also not significantly different between the HFPEF and HFREF groups (Table 1).

**Table 1:** Baseline clinical data.

Demographics	HC	HFPEF	HFREF	p-value
Number	50	100	100	---
Age, years	54 (52–62)	72 (63–79)	65 (59–73)	<0.001
Sex: male, %	48	62	71	0.029
Race: white, %	86	85	90	0.701
Physical Characteristics				
Obese, %	22	59	42	<0.001
Systolic BP	122 (115–136)	128 (118–141)	119 (104–132)	0.005
Diastolic BP	74 (67–78)	71 (63–79)	72 (64–80)	0.449
Medical History				
Smoker, %	18	61	52	<0.001
HTN, %	N/A	78	60	0.006
DM, %	N/A	46	36	0.134
PVD, %	0	10	5	0.071
AFib, %	N/A	52	40	0.089
NYHA Class, %				0.072
I	N/A	12	22	
II	N/A	56	47	
III	N/A	32	28	
IV	N/A	0	3	
Primary Etiology of HF, %				<0.001
Ischemic	N/A	14	37	
Non-ischemic	N/A	86	63	
Laboratory Values				
BNP, pg/mL	16 (11–28)	76 (44–236)	162 (79–398)	<0.001
SrCR, µM	72 (59–85)	99 (79–138)	96 (82–116)	<0.001
Total Cholesterol, mM	5.4 (4.8–5.8)	3.8 (3.2–4.4)	3.6 (3.0–4.4)	<0.001
Triglycerides, mM	1.3 (0.8–1.6)	1.2 (0.9–1.8)	1.3 (0.9–2.4)	0.662
Cholesterol: HDL Ratio	3.9 (3.1–4.5)	3.3 (2.8–4.3)	3.6 (2.9–4.5)	0.276
eGFR, mL/min/1.73 m^2^	76 (62–85)	57 (41–78)	59 (48–72)	<0.001
Medication				
Antiarrhythmic, %	N/A	11	7	0.323
ACEi or ARB, %	N/A	86	89	0.521
Beta-blocker, %	N/A	30	36	0.367
Digoxin, %	N/A	11	16	0.301
Loop diuretic, %	N/A	78	68	0.111
MRA, %	N/A	19	38	0.003
NSAIDs, %	0	8	8	0.118
Thiazide diuretic, %	N/A	12	7	0.228
Statin, %	2	73	68	<0.001

Abbreviations: HC, healthy control; HFPEF, heart failure (HF) with preserved ejection fraction; HFREF, HF with reduced ejection fraction; BP, blood pressure; HTN, hypertension; DM, diabetes; PVD, peripheral vascular disease; AFib, history of atrial fibrillation; NYHA, New York Heart Association; BNP, B-type natriuretic peptide; SrCr, serum creatinine; HDL, high-density lipoprotein; eGFR, estimated glomerular filtration rate; ACEi, angiotensin converting enzyme inhibitor; ARB, angiotensin II receptor antagonist; MRA, mineralocorticoid receptor antagonist; and NSAIDs, non-steroidal anti-inflammatory drugs. See Methods section for details on how parameters were obtained. P-value represents Mann-Whitney U Test, Kruskal-Wallis Test or Chi-square Test where appropriate. Number was not tested, as the sample sizes were selected *a priori*.

Heart rates did not differ significantly between controls or the two HF groups; however, a significantly greater number of HFPEF patients were in AFib on the day of study than HFREF patients (p = 0.018; Table 2). The HFREF group had significantly lower LVEF compared to control and HFPEF groups, while both HFPEF and HFREF had greater LV posterior wall thickness (LVPW; p<0.001 for both) and mass index (LVMI; p<0.001 for both); left atrial volume index (LA volume index; p<0.001 for both); and average E/e' ratio (p<0.001 for both) compared to control (Table 2). LVPW (p<0.05), and left ventricular end diastolic and systolic dimensions (LVEDD and LVESD, respectively; p<0.001 for both) were significantly different between the two HF groups (Table 2). The distributions of grades of diastolic dysfunction were also significantly different (p = 0.043), which might be due to the high prevalence of AFib acting as a confounder in this analysis (Table 2). Significantly more HFREF subjects had left-ventricular hypertrophy (LVH) than HFPEF subjects (p<0.001; Table 2).

**Table 2:** Electrocardiogram and Echocardiography.

	HC	HFPEF	HFREF	p-value
HR, bpm	65 (60–76)	65 (60–78)	65 (60–76)	0.757
AFib, %	0	43	23	<0.001
LVEF, %	63 (60–67)	59 (54–63)	35 (27–41)	<0.001
LVEDD, cm	4.4 (4.1–4.6)	4.8 (4.3–5.2)	5.9 (5.4–6.4)	<0.001
LVESD, cm	2.8 (2.5–3.2)	3.1 (2.8–3.6)	4.7 (3.8–5.6)	<0.001
LVPW, cm	0.9 (0.8–1.0)	1.1 (1.0–1.2)	1.0 (0.9–1.1)	<0.001
LVMI, g/m^2^				
Female	62 (54–69)	89 (78–111)	103 (80–119)	<0.001
Male	74 (56–87)	105 (83–119)	129 (104–152)	<0.001
LVH, %	6	34	60	<0.001
LA index, mL/m^2^	23 (19–27)	34 (28–43)	37 (30–50)	<0.001
MR, %				<0.001
None	38	45	32	
Trace/mild	12	43	41	
Moderate	0	11	20	
Severe	0	1	7	
E-wave velocity, cm/s	74 (67–82)	87 (73–107)	76 (60–97)	<0.001
Medial E/e' ratio	9 (8–11)	14 (10–17)	15 (11–20)	<0.001
Lateral E/e' ratio	7 (6–8)	10 (8–13)	11 (8–16)	<0.001
Average E/e' ratio	8 (7–10)	12 (9–15)	13 (10–18)	<0.001
A-wave velocity, cm/s	68 (61–78)	79 (63–96)	74 (55–89)	0.052
E/A ratio	1.0 (0.8–1.2)	0.9 (0.8–1.3)	1.0 (0.6–1.6)	0.745
Grade: diastolic dysfunction*, %				<0.001
0 (Normal)	94	34	14	
1 (Impaired relaxation)	4	25	31	
2 (Pseudonormal filling)	2	24	34	
3 (Restrictive filling)	0	17	21	

Abbreviations: HC, healthy control; HFPEF, heart failure (HF) with preserved ejection fraction; HFREF, HF with reduced ejection fraction; HR, heart rate; AFib, atrial fibrillation based on ECG; LVEF, left-ventricular ejection fraction; LVEDD, left-ventricule (LV) end diastolic diameter; LVESD, LV end systolic diameter; LVPW, LV posterior wall thickness; LVMI, LV mass indexed to body surface area (BSA); LVH, left-ventricular hypertrophy; LA index, left-atrial volume indexed to BSA; MR, mitral regurgitation; E-wave, early diastolic wave velocity; e', mitral valve annular velocity as measured medially or laterally by way of tissue Doppler imaging; and A-wave, late diastolic velocity due to atrial systole. See Methods section for details on how parameters were obtained. P-value represents Kruskal-Wallis Test or Chi-square Test where appropriate.*Grade of diastolic dysfunction excluding those patients with severe MR or poor visualization on echocardiography.

### Circulating inflammatory markers in healthy control, HFPEF and HFREF

**Figure 1:**
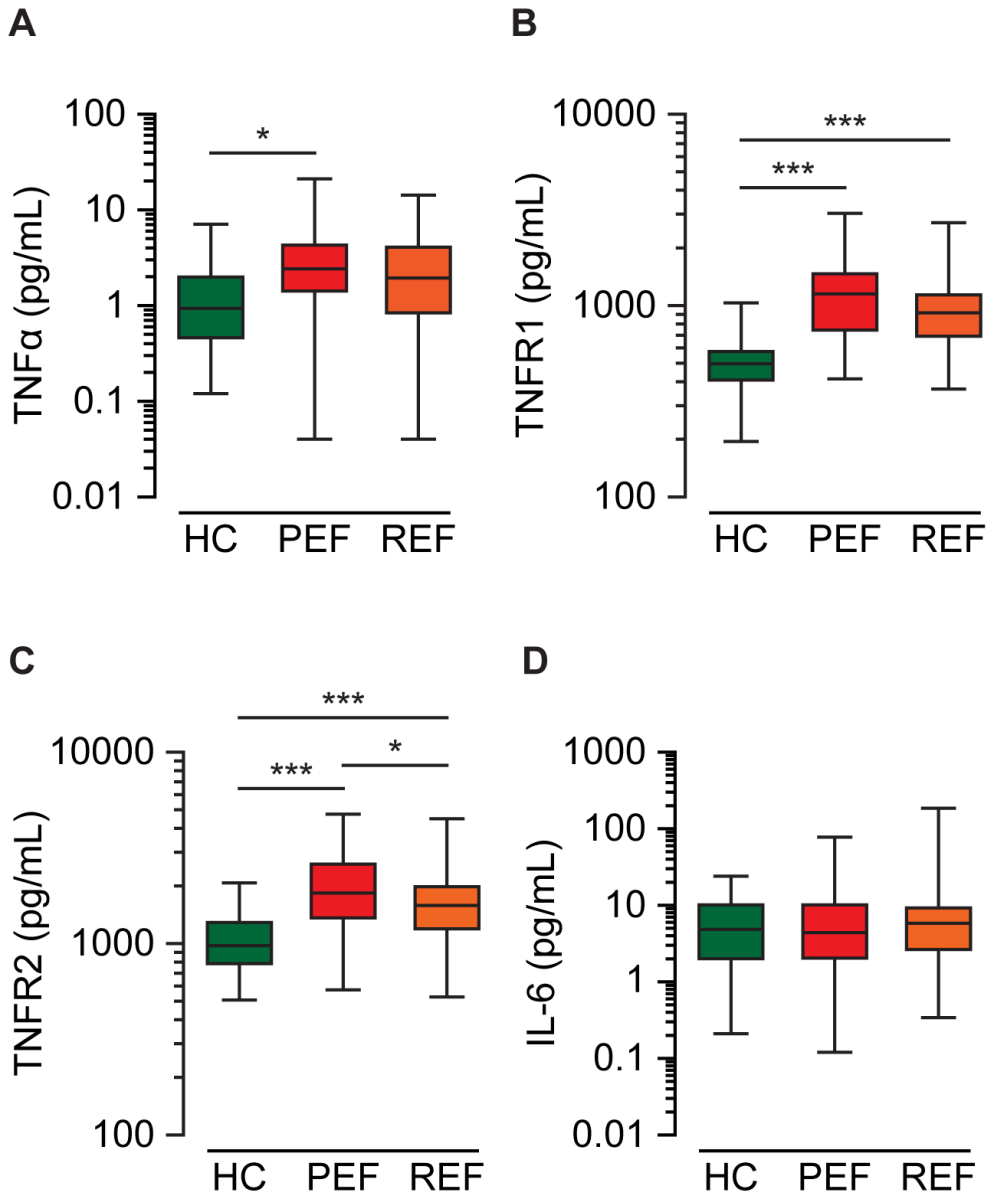
Circulating inflammatory markers in healthy control (HC), HFPEF (PEF) and HFREF (REF). Box and whisker plots show the relative distributions of TNF-α (A), TNFR1 (B), TNFR2 (C) and IL-6 (D) levels. * P<0.05, *** P<0.001 for Kruskal-Wallis Test with pairwise comparisons.

Compared to control, plasma levels of TNFα were only significantly elevated in HPFEF, while levels of TNFR1 and TNFR2 were significantly elevated in both HFPEF and HFREF (Figure 1A–C). Meanwhile, IL-6 was not significantly different between control and the HF groups (Figure 1D). Interestingly, TNFR2 was significantly elevated in HFPEF relative to HFREF, while TNFα and TNFR1 were non-significantly elevated in HFPEF (Figure 1A–C). We explored the relationship between age, eGFR, sex, obesity, LVH, hypertension, diabetes, smoking status, peripheral vascular disease or history of atrial fibrillation with TNFα, TNFR1, TNFR2 and IL-6 in the combined HF group (Table 3). Advanced age was significantly associated with elevated TNFR1 and TNFR2, and low eGFR was significantly associated with elevated TNFα in addition to its two receptors (Table 3). TNFα, TNFR1 and TNFR2 increased differentially in response to smoking status or comorbid conditions, such as hypertension and diabetes, but were always associated with at least two covariates, while IL-6 was not associated with any covariate (Table 3). Sex, obesity and LVH were not significantly associated with any of the inflammatory markers (Table 3).

**Table 3:** Univariate correlations of biomarkers with continuous and binary covariates.

	Marker											
	TNFα			TNFR1			TNFR2			IL-6		
Continuous	R	p-value		R	p-value		R	p-value		R	p-value	
Age	0.077	0.331		0.204	0.004		0.161	0.023		0.038	0.617	
eGFR	0.187	0.020		0.534	<0.001		0.433	<0.001		0.082	0.280	

Binary	OR (95%CI)	p-value	OR (95%CI)	p-value	OR (95%CI)	p-value	OR (95%CI)	p-value
Sex	1.68 (0.87–3.23)	0.121	0.60 (0.13–2.76)	0.513	0.50 (0.11–2.26)	0.368	1.42 (0.78–2.57)	0.251
Obese	1.40 (0.75–2.62)	0.294	2.27 (0.54–9.60)	0.266	1.84 (0.45–7.60)	0.397	1.17 (0.67–2.05)	0.578
LVH	1.68 (0.84–3.36)	0.142	0.69 (0.16–2.99)	0.622	0.46 (0.11–1.95)	0.290	1.13 (0.64–1.99)	0.677
HTN	2.04 (1.04–4.00)	0.038	11.59 (2.21–60.75)	0.004	3.24 (0.69–15.18)	0.136	0.64 (0.34–1.19)	0.162
DM	1.58 (0.82–3.04)	0.172	20.21 (4.05–100.96)	<0.001	11.29 (2.42–52.75)	0.002	0.97 (0.55–1.71)	0.918
Smoker	2.00 (1.04–3.82)	0.037	1.68 (0.39–7.13)	0.485	3.57 (0.84–15.24)	0.085	0.95 (0.54–1.67)	0.857
PVD	1.04 (0.35–3.12)	0.944	23.63 (1.30–430.17)	0.037	11.78 (0.68–204.67)	0.090	1.04 (0.37–2.92)	0.942
AFib	1.97 (1.01–3.81)	0.045	4.09 (0.94–17.77)	0.060	4.81 (1.12–20.74)	0.035	0.90 (0.52–1.58)	0.716

Abbreviations: eGFR, estimated glomerular filtration rate; LVH, left-ventricular hypertrophy; HTN, hypertension; DM, diabetes mellitus; PVD, peripheral vascular disease; and AFib, history of atrial fibrillation. See Methods section for details on how parameters were obtained.

### Modulators of inflammatory markers in HFPEF and HFREF

**Figure 2:**
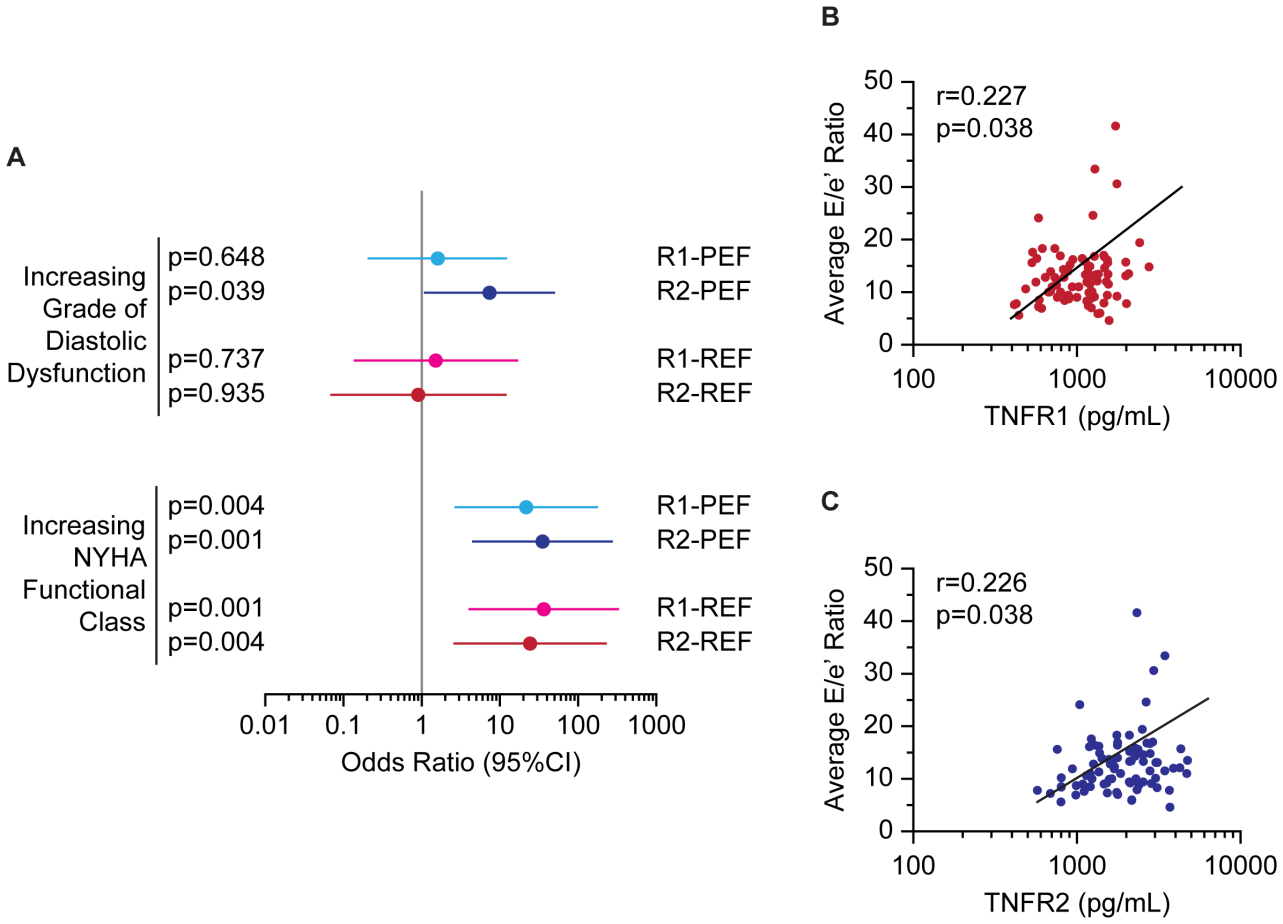
Associations of TNFR1 and TNFR2 with disease parameters. Forest plots show odds ratios and 95% confidence intervals (95%CI) for TNFR1 (R1) or TNFR2 (R2) as predictors of increasing grade of diastolic dysfunction or NYHA class in HFPEF (PEF) or HFREF (REF) (A). Scatterplots show average E/e' ratio as a function of TNFR1 (B) or TNFR2 (C) in HFPEF.

We investigated how the inflammatory markers in this study related to two clinically-relevant and commonly-used ordinal quantifications of disease status in HF: grade of diastolic dysfunction and NYHA functional class. We investigated these associations using ordinal logistic regression analyses. Elevated TNFR2 was significantly associated with increasing grade of diastolic dysfunction in HFPEF, but not in HFREF, while TNFR1 was not associated with diastolic dysfunction in either HF group (Figure 2A). Interestingly, elevated TNFR1 and TNFR2 levels were significantly associated with increasing NYHA functional class in both HPFEF and HFREF (Figure 2A). Neither TNFα, nor IL-6 were significantly associated with diastolic dysfunction or symptom severity in either HFPEF or HFREF (data not shown). LA volume index was not associated with plasma TNFR1 or TNFR2 levels in HFPEF (r = 0.110, p = 0.298; and r = 0.170, p = 0.105, respectively) or HFREF (r = 0.083, p = 0.443; and r = 0.056, p = 0.607, respectively). Plasma levels of TNFR1 and TNFR2 were weakly, but significantly, associated with average E/e' ratio in HFPEF (Figure 2B & C), but not HFREF (r = 0.215, p = 0.053; and r = 0.112, p = 0.318, respectively). Given that AFib can confound diastolic dysfunction analyses, we investigated associations between TNFR1 or TNFR2 and LA volume index or average E/e' ratio, as alternative measures of diastolic function that can be used for patients in AFib [25].

**Figure 3:**
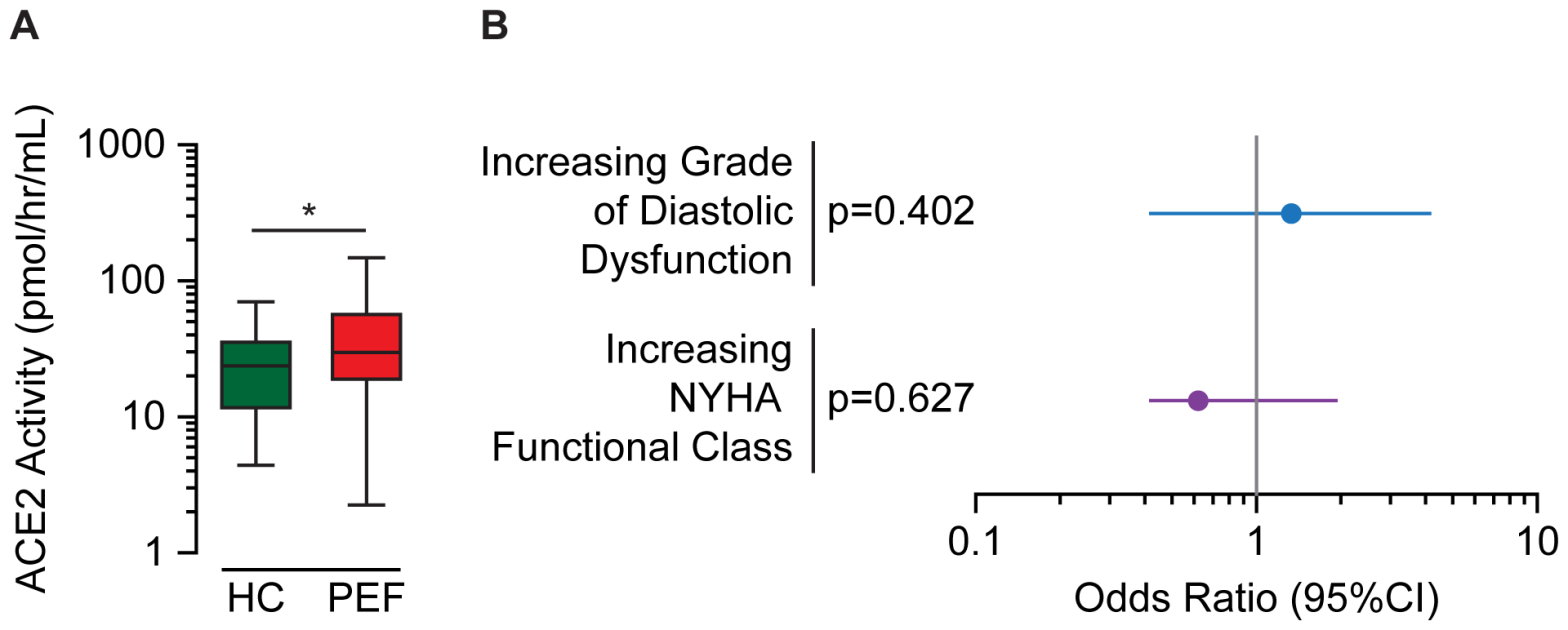
Angiotensin-converting enzyme 2 (ACE2) activity in HFPEF, and comparison with disease parameters. Box and whisker plots compare the distribution of plasma ACE2 activity in healthy control (HC) and HFPEF (PEF) (A). Forest plots show odds ratio and 95% confidence interval (95%CI) for ACE2 as predictor of increasing grade of diastolic dysfunction or NYHA class in HFPEF (B). * P<0.05 for Mann Whitney U test.

Increased activity of TNFα converting enzyme (TACE), a sheddase involved in proteolytic processing of TNFα, TNFRs, and angiotensin-converting enzyme 2 (ACE2) might represent a mechanism of increased circulating TNFα and TNFRs in HF [26], [27]. ACE2 is a counter-regulatory homologue of angiotensin-converting enzyme (ACE) and a major regulator of endothelial function and myocardial fibrosis [28]–[30]. Since the membrane-bound localization of TACE poses a limitation in assessing its levels or activity in the plasma, we measured plasma ACE2 activity as a surrogate, as it increases with increased TACE activity [31]. Previous reports of plasma ACE2 activity showed an association with clinically diagnosed HFREF, symptom severity and worsening clinical outcomes [20], [32]. In our HFPEF cohort, plasma ACE2 activity was significantly elevated relative to control (Figure 3A); however, it was not associated with increasing grade of diastolic dysfunction or NYHA class (Figure 3B).

## Discussion

In this comparative analysis of healthy controls, and HF patients with preserved or reduced left-ventricular ejection fraction, we found that our cohorts recapitulated previous characterizations, whereby, HFPEF subjects were more likely to be older, hypertensive and obese [3]–[5], [24]. Similarly, AFib was prevalent in both HF groups, which follows the trend observed in the MAGGIC meta-analysis [5]. Furthermore, the primary etiology of HF was much less likely to be ischemic in the HFPEF group. Despite differences in comorbidities, age and HF etiology the NYHA class distribution was similar between HFREF and HFPEF, which indicates that the two patient populations experienced significant burden of disease. Additionally, both groups had cardiac hypertrophy and exhibited marked diastolic dysfunction. In patients without marked systolic dysfunction, a greater prevalence of comorbidities might account for the similarity in symptoms between HFPEF and HFREF.

Improvement in exercise capacity in a cohort of HFPEF patients after an exercise training regimen was largely mediated by peripheral improvements, which suggests a systemic component to HFPEF, including skeletal muscle dysregulation [33]. Indeed, a recent model of HFPEF suggests that comorbidity-mediated systemic inflammation, results in coronary microvascular endothelial dysfunction, myocardial hypertrophy and fibrosis, and skeletal muscle dysregulation to produce the HFPEF phenotype [6]. We investigated TNFα and its receptors (TNFR1 and TNFR2) as one component of comorbidity-driven inflammation, and included IL-6 as non-TNF-family cytokine for comparison. We expand on previous work in HF: our data showed that levels of TNFα and TNFR1 were significantly increased in HFPEF relative control but not HFREF, while TNFR2 was significantly increased relative to both control and HFREF [14]. Plasma IL-6 levels were very comparable between HFPEF and HFREF, which suggests that TNFα-mediated inflammation might be a point of pathophysiological difference between HF phenotypes.

We found that low eGFR, hypertension, smoking status and history of atrial fibrillation were significantly associated with elevated TNFα levels, while elevated TNFR1 levels were associated with aging, low eGFR, hypertension, diabetes and PVD, and elevated TNFR2 levels were associated with aging, low eGFR, diabetes and atrial fibrillation. The advanced age and greater prevalence of comorbidities in the HFPEF population could drive the observed elevation in circulating inflammatory markers relative to HFREF. Indeed, non-cardiac comorbidities are more prevalent in HFPEF, with a larger fraction of adverse clinical outcomes attributable to non-cardiac events compared to HFREF [34]. Likewise, increased TNFα-axis inflammation has been linked to an increased risk of adverse cardiovascular and all-cause outcomes in HF [18], [35], [36]. Altogether, these data are congruent with the paradigm for HFPEF that includes comorbidities-driven dysregulation of TNFα-mediated signaling [6], [34].

Our finding that circulating TNFR2 levels are significantly associated with increasing average E/e' ratio and grade of diastolic dysfunction in HFPEF, but not in HFREF, suggests a greater role for TNFα-mediated inflammation in this cohort. However, we found that TNFR1 and TNFR2 levels were significantly associated with increasing NYHA class in both HF phenotypes, which shows that part of the HFREF phenotype can also be attributed to inflammation. The association of diastolic dysfunction and symptoms with TNFR2 in HFPEF is consistent with findings in experimental models, as the TNFR1/TNFR2 axis is involved in mediating divergent effects: TNFR1 has been implicated in adverse cardiac remodeling and adipogenesis, while TNFR2 antagonizes the pathological effects of TNFR1, and also stimulates angiogenesis [37]–[40]. TNFR1 and TNFR2 also mediate opposite effects on phospholamban and SERCA2, key Ca^2+^ handling proteins involved in myocardial relaxation [37], [41]. An increase in circulating TNFR2 levels might reflect a loss of protective signaling mechanisms due to tissue shedding of TNFR2, thereby leading to the correlation between circulating TNFR2 levels and diastolic dysfunction and symptoms in HFPEF.

The differential role of TNFRs might explain why TNFα is not associated with function or symptoms: the variable response to TNFα is mediated at the receptor level. Congruent with a recent report, we found TNFα was not strongly associated with either HF phenotype; however, Marti *et al.* implicate TNFR1, while our study implicates TNFR2 in HFPEF [13]. The key difference between the two studies is that Marti *et al.* explored risk of incident HF, while we examined existing HF populations. Indeed, we found TNFR1 to be associated with the greatest number of HF risk factors, which is congruent with a role for TNFR1 in precipitating incident HFPEF. Meanwhile, our data show that TNFR2 might then be a better biomarker for gauging severity of established HFPEF.

Circulating fragments of TNFα, TNFR1 and TNFR2 are generated from full, transmembrane proteins through the activity of TACE [26], [27]. We hypothesized that increased circulating levels of TNFR1 and TNFR2 might reflect increased activity of TACE. ACE2 is also a substrate of TACE, and plasma ACE2 activity increases in conjunction with increased TACE activity [26], [31]. Since TACE is membrane-bound, as a surrogate of TACE-activity we measured plasma ACE2 activity in HFPEF patients relative to healthy controls. Previous work showed a significant association between ACE2 and symptom severity and adverse clinical outcomes in patients with HFREF [20], [32]. We found significantly elevated plasma ACE2 activity in HFPEF as compared to healthy controls, but no association to diastolic dysfunction or symptoms. This is congruent with the idea that elevated ACE2 activity is a consequence of the drivers of HFPEF, rather than an effector as may be the case in HFREF [20], [32].

## Conclusion

Taking our data together with previous reports indicates that the TACE/TNFα/TNFR1/TNFR2 inflammatory axis is a part of the pathogenesis of HFPEF [7]–[10], [37]–[40]. In the context of the failure of TNFα antagonism as a therapeutic tool in HF, our data suggest that a downstream approach involving TNFR1 inhibition or TNFR2 potentiation may represent a more effective therapeutic approach for patients with HFPEF [42].

## Limitations

Our study, while larger than most other studies that compared levels of TNFα and its receptors in HFPEF versus HFREF, is still relatively small in terms of clinical studies. Indeed, TNFα might also be informative with respect to differentiating HFPEF from HFREF, but our study cannot resolve whether this is the case. In addition, the results reported herein represent a single cross-section across healthy controls, and HFPEF and HFREF patients, so follow-up measurements are not included, nor are outcomes data. Further studies with a longitudinal component will be important for implicating various molecules or pathways in the pathophysiological development of HF, for which work by Marti *et al.* is a strong contribution [13]. Finally, our results only provide correlative evidence of whether these biochemical markers are primary mediators of the HF syndrome, or end-effectors of pathogenic processes. Indeed, while a conglomerate of evidence points towards an interplay between plasma and tissue levels for TNFR1 and TNFR2—shedding increases plasma levels at the expense of tissue levels—direct tissue and plasma comparisons from the same subjects will be necessary to determine the relative effects of proteolytic processing and changes in expression.
